# Smartphone App–Based Assessment of Gait During Normal and Dual-Task Walking: Demonstration of Validity and Reliability

**DOI:** 10.2196/mhealth.8815

**Published:** 2018-01-30

**Authors:** Brad Manor, Wanting Yu, Hao Zhu, Rachel Harrison, On-Yee Lo, Lewis Lipsitz, Thomas Travison, Alvaro Pascual-Leone, Junhong Zhou

**Affiliations:** ^1^ Hebrew SeniorLife Institute for Aging Research Harvard Medical School Roslindale, MA United States; ^2^ Berenson-Allen Center for Noninvasive Brain Stimulation Division of Interventional Cognitive Neurology Beth Israel Deaconess Medical Center Boston, MA United States

**Keywords:** smartphone, gait assessment, pocket, dual task, validity, reliability, mobile applications

## Abstract

**Background:**

Walking is a complex cognitive motor task that is commonly completed while performing another task such as talking or making decisions. Gait assessments performed under normal and “dual-task” walking conditions thus provide important insights into health. Such assessments, however, are limited primarily to laboratory-based settings.

**Objective:**

The objective of our study was to create and test a smartphone-based assessment of normal and dual-task walking for use in nonlaboratory settings.

**Methods:**

We created an iPhone app that used the phone’s motion sensors to record movements during walking under normal conditions and while performing a serial-subtraction dual task, with the phone placed in the user’s pants pocket. The app provided the user with multimedia instructions before and during the assessment. Acquired data were automatically uploaded to a cloud-based server for offline analyses. A total of 14 healthy adults completed 2 laboratory visits separated by 1 week. On each visit, they used the app to complete three 45-second trials each of normal and dual-task walking. Kinematic data were collected with the app and a gold-standard–instrumented GAITRite mat. Participants also used the app to complete normal and dual-task walking trials within their homes on 3 separate days. Within laboratory-based trials, GAITRite-derived heel strikes and toe-offs of the phone-side leg aligned with smartphone acceleration extrema, following filtering and rotation to the earth coordinate system. We derived stride times—a clinically meaningful metric of locomotor control—from GAITRite and app data, for all strides occurring over the GAITRite mat. We calculated stride times and the dual-task cost to the average stride time (ie, percentage change from normal to dual-task conditions) from both measurement devices. We calculated similar metrics from home-based app data. For these trials, periods of potential turning were identified via custom-developed algorithms and omitted from stride-time analyses.

**Results:**

Across all detected strides in the laboratory, stride times derived from the app and GAITRite mat were highly correlated (*P*<.001, *r*^2^=.98). These correlations were independent of walking condition and pocket tightness. App- and GAITRite-derived stride-time dual-task costs were also highly correlated (*P*<.001, *r*^2^=.95). The error of app-derived stride times (mean 16.9, SD 9.0 ms) was unaffected by the magnitude of stride time, walking condition, or pocket tightness. For both normal and dual-task trials, average stride times derived from app walking trials demonstrated excellent test-retest reliability within and between both laboratory and home-based assessments (intraclass correlation coefficient range .82-.94).

**Conclusions:**

The iPhone app we created enabled valid and reliable assessment of stride timing—with the smartphone in the pocket—during both normal and dual-task walking and within both laboratory and nonlaboratory environments. Additional work is warranted to expand the functionality of this tool to older adults and other patient populations.

## Introduction

Walking is central to many activities of daily living and is most typically completed while simultaneously performing unrelated cognitive tasks, for example, talking, reading signs, or making decisions. Even in healthy adults, such dual tasking reduces gait speed, prolongs stride time, and increases stride-to-stride movement variability [1]. These performance decrements, or “costs,” indicate that walking is regulated by a complex control system dependent on numerous cognitive functions and underlying brain networks. Therefore, the assessment of gait under normal and dual-task conditions provides valuable insights into not only one’s physical health [2,3], but also one’s brain health, and even the likelihood of developing dementia several years into the future [4,5].

Gait assessments are typically completed within clinical or laboratory settings. They are thus inaccessible to those living in remote settings and do not lend themselves well to high-frequency monitoring. Moreover, clinical assessments entail qualitative evaluation, are predisposed to subjective bias, and are often insensitive to subtle gait disturbances [6-9]. Laboratory assessments overcome these limitations by quantifying temporospatial characteristics of gait, yet they require expensive equipment, dedicated laboratory space, and trained personnel. There is thus an urgent need to develop mobile tools that enable low-cost quantitative assessments of gait.

Smartphones contain a 3-dimensional accelerometer, a 3-dimensional gyroscope, and a digital compass that are similar in sensitivity to research-grade biomechanical instrumentation. The smartphone, when secured to an individual’s lower back or sternum as they walk, can detect gait events such as heel strikes [10], as well as kinematic differences between those with and those without movement disorders, such as Parkinson disease [11,12]. Still, studies to date have been limited to laboratory environments and have required trained personnel to administer assessments, provide instructions, and secure the phone to the participant’s trunk.

In collaboration with Sage Bionetworks (Seattle, WA, USA) and supported by the Football Players Health Study at Harvard University, the objective of this study was to create an iPhone-based app enabling the administration of a standardized gait assessment, under both normal and dual-task conditions, within nonlaboratory settings. The app was designed to provide multimedia instructions to the user, acquire data with the phone placed in the user’s pocket, and derive stride times from bouts of walking. We chose stride time because it can be directly derived from gait events (eg, heel strikes), is closely linked to gait speed [13], and has been associated with aging [14], movement disorders [15], cognitive impairment [16], and the development of falls [17]. As turning significantly disrupts stride timing [18], we also developed a method of automatically detecting turns. We determined the validity and reliability of the app by (1) comparing the accuracy of stride times derived from the app versus those derived from gold-standard laboratory instrumentation, and (2) determining the test-retest reliability of app-derived stride times within both laboratory- and home-based settings.

## Methods

### Smartphone App

**Figure 1:**
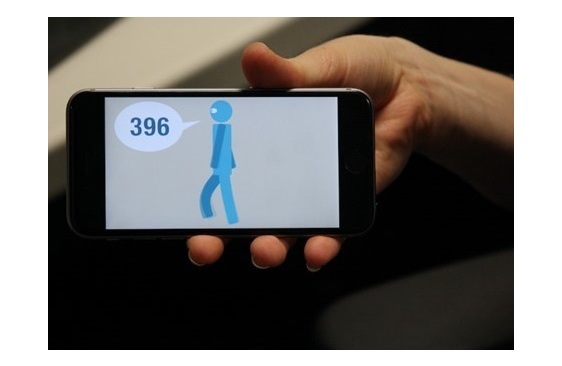
Screenshot of the animated instruction for dual-task walking. The app provides text and animated instructions prior to the assessment, followed by voice instructions during the assessment, to enable gait analysis from data acquired while the user walks with their phone placed in the pocket of their pants or shorts. Gait is assessed while individuals walk normally, and again while they walk and simultaneously perform a serial-subtraction cognitive dual task.

The app was designed to recreate a common laboratory-based dual-task gait assessment, namely, evaluation of walking under normal conditions and again while verbalizing serial subtractions of 3 from a random number between 200 and 999 [19,20]. The app provided multimedia instructions to the participant to help ensure reliability of results. Participants first watched an animation developed by Wondros Inc (Los Angeles, CA, USA) that provided a general overview of the assessment (Figure 1). The user was then presented with several on-screen text instructions. The last page instructed the participant to press Start and place the phone in their preferred front pocket. The iPhone speaker was then used to provide audible instructions to the participant for the remainder of the assessment. These instructions provided the procedural details of each walking trial, cues for the start and end of each trial, and, for dual-task trials, a randomly generated starting number for the serial-subtraction task.

We designed walking assessments to include one 45-second trial of normal walking and one 45-second trial of dual-task walking. Trial start and end cues triggered acquisition of accelerometer, gyroscope, and magnetometer data, which were stored on the phone’s internal storage capacity. Following each assessment, the participant was prompted to answer a multiple-choice question (see following section) presented in text format on the smartphone screen. Kinematic and questionnaire data were then automatically uploaded via Wi–Fi to a remote, cloud-based data server for offline analyses.

### Participants

We recruited men and women aged 18 to 35 years via local advertisement. Exclusion criteria were an inability to walk unassisted; self-report of major disease, such as stroke, Parkinson disease, diabetes mellitus, or cardiovascular disease; history or presence of ulceration, amputation, or other painful symptoms in the lower extremities; drug or alcohol abuse; and hospitalization within the past 6 months. Interested and eligible individuals provided written informed consent as approved by the Hebrew SeniorLife Institutional Review Board (Hebrew SeniorLife Institute for Aging Research, Roslindale, MA, USA; approval number: IRB-2015-40).

### Study Procedures

Participants completed 2 laboratory visits separated by 1 week. They completed the same assessments of locomotor control within each visit, during which data were simultaneously collected via the app and a 14-foot instrumented GAITRite mat (CIR Systems, Inc, Franklin, NJ, USA). Participants additionally used the app to complete walking assessments within their homes on 3 separate days, in between their 2 laboratory visits. No instructions were provided regarding time of day for home assessment completion.

#### Laboratory Assessments

Participants completed 2 laboratory visits separated by at least one week. We instructed them to wear comfortable shoes and pants or shorts with front pockets for each visit. The same procedures were completed on each visit to enable testing of between-visit test-retest reliability. Within each visit, participants completed the app walking assessment 3 separate times, such that they completed 3 pairs of normal walking and dual-task walking trials. Trial order was randomized with each pair.

Within the laboratory, each walking trial was completed around an oval-shaped, 24-m indoor track. We placed the GAITRite mat along one long side of the track. Each trial began with participants standing just behind the beginning of the mat to ensure that the first footfall of each trial was captured by the mat. Participants used the app instructions to initiate and complete each trial.

After all trials, the app prompted participants to use the iPhone touch screen to answer the following multiple-choice question: “How tight is the pocket in which you placed the phone? (tight, medium or loose).” We did this to study the effects of this variable on the ability to collect valid and reliable data over time. The questionnaire was incorporated into the app using the SageBridge online portal (Sage Bionetworks).

#### Home Assessments

We asked participants to use the app to complete a walking assessment (1 normal walk and 1 dual-task walk) at home on 3 separate days in between their laboratory visits. The app provided the same instructions to the participant as during the laboratory visit. Additionally, the app instructed participants to complete the walk in a quiet room or hallway, and to walk continuously throughout the trial, making turns if and when needed. On completion of both trials, participants were prompted to answer the same multiple-choice question regarding pocket tightness as described in the laboratory assessment.

### Data Analysis

#### Laboratory Assessments

**Figure 2:**
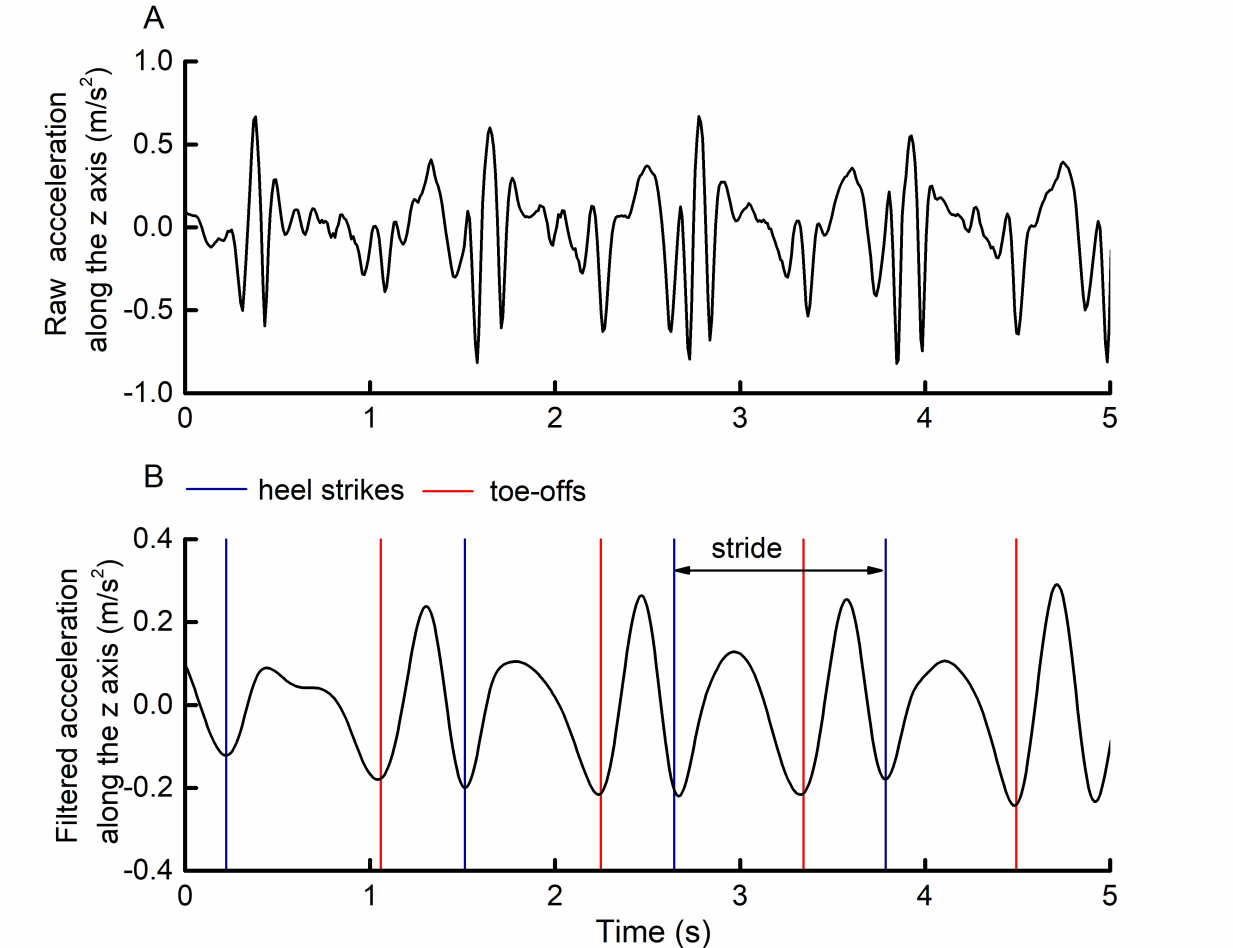
Example of (A) raw and (B) filtered smartphone-recorded accelerations along the earth coordinate system vertical axis during straight walking, relative to identified gait events. Phone-side leg heel-strike and toe-off events derived from a GAITRite mat were overlaid on vertical-axis accelerations acquired by a smartphone placed in the participant’s pocket. These heel-strike and toe-off events correspond to trough nadirs following relatively high peaks and low peaks, respectively, within the filtered acceleration time series.

The app sampled kinematic data at a frequency of 100 Hz. Raw, 3-dimensional accelerometer and gyroscope time series were each transformed from the device coordinate system to an earth coordinate system using the quaternion rotation matrix. Following this rotation, the z-axis formed a line between the center of the earth and the phone, and was thus approximately vertical to the ground (see Figure 2, part A, for example acceleration data). Each rotated z-axis time series was then filtered with a common Butterworth filter. These time series, which contained peaks that alternated between relatively high and low amplitudes, aligned with heel-strike and toe-off events derived from the GAITRite mat (Figure 2, part B). Specifically, each heel strike corresponded to the trough nadir following each relatively high peak, whereas toe-offs corresponded to the trough nadir following each relatively low peak.

We defined stride time as the time elapsed between 2 consecutive heel strikes of the same foot. We calculated it by determining the number of data points between 2 consecutive trough nadirs following relatively high peaks, and then dividing by the sampling frequency of 100 Hz. For all strides that took place on the GAITRite mat, we calculated stride times from both gait mat and app data and used these for analyses.

#### Automatic Turn Detection

**Figure 3:**
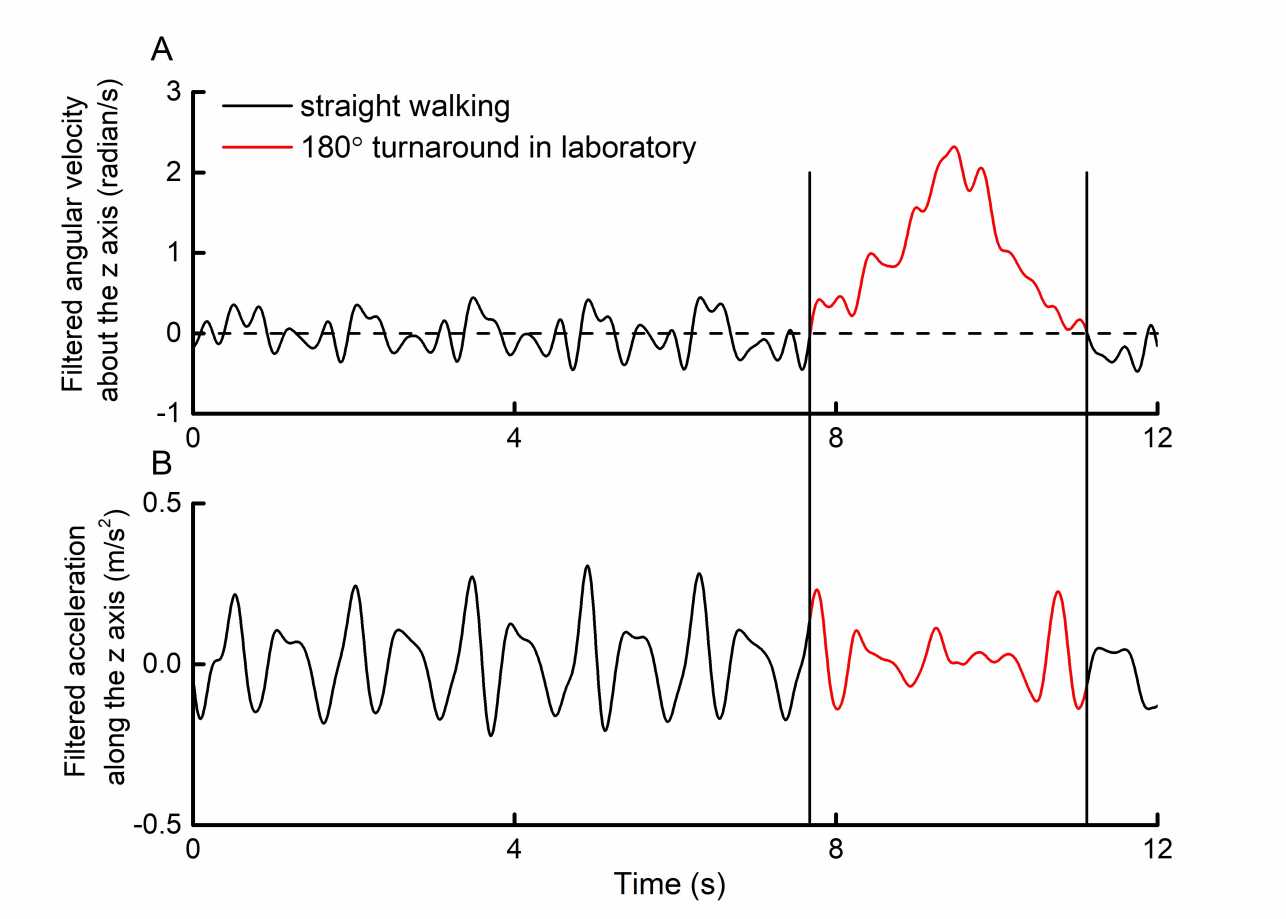
Example of smartphone-recorded (A) angular velocity and (B) acceleration relative to the earth coordinate system vertical axis during straight walking and a 180° turn. In a pilot trial, a participant walked straight across the laboratory before turning around a cone. Angular velocities were relatively small and contained numerous zero crossings during the straight-walking portion of the trial. Turning, on the other hand, was associated with a large nonstationarity in angular velocity between the 2 adjacent zero crossings. Acceleration patterns were noticeably altered during this period. This observation was subsequently used to develop a method to identify potential turns from walking trials collected during in-home assessments.

Walking trials completed at home likely included variable amounts of turning. Turning, while itself an important functional measure, alters stride timing [18]. We therefore developed a method to identify relatively sharp, rapid turns to enable stride-time calculation from bouts of walking without such turns. Turning produces a large deviation in the angular velocity about the body’s vertical axis. In pilot studies of straight-line walking with the phone placed in the pocket, z-axis gyroscope data contained fluctuations of relatively small amplitude with frequent zero crossings (Figure 3, part A, black line portions). However, during a 180° turn, this angular velocity was significantly greater in 1 direction—depending on the direction of turning relative to the phone’s orientation in the pocket—with no zero crossings (see Figure 3, part A, red line portion).

The total angular distance traveled in 1 direction can be calculated by integrating the angular velocity time series between 2 consecutive zero crossings (ie, area under the curve [AUC]). A 180° turn of the phone’s gyroscope would thus equal 3.14 radians (ie, π). The AUC related to the 180° turn depicted in Figure 3, part A, was 3.37. We also noted in our unpublished pilot studies that relatively rapid turns were less likely to contain higher-frequency angular velocity fluctuations that crossed zero. For this analysis, we therefore defined a turn as any period between 2 consecutive zero crossings within rotated, filtered z-axis angular velocity time series in which the product of the AUC and the time between zero crossings eclipsed a predefined threshold.

**Figure 4:**
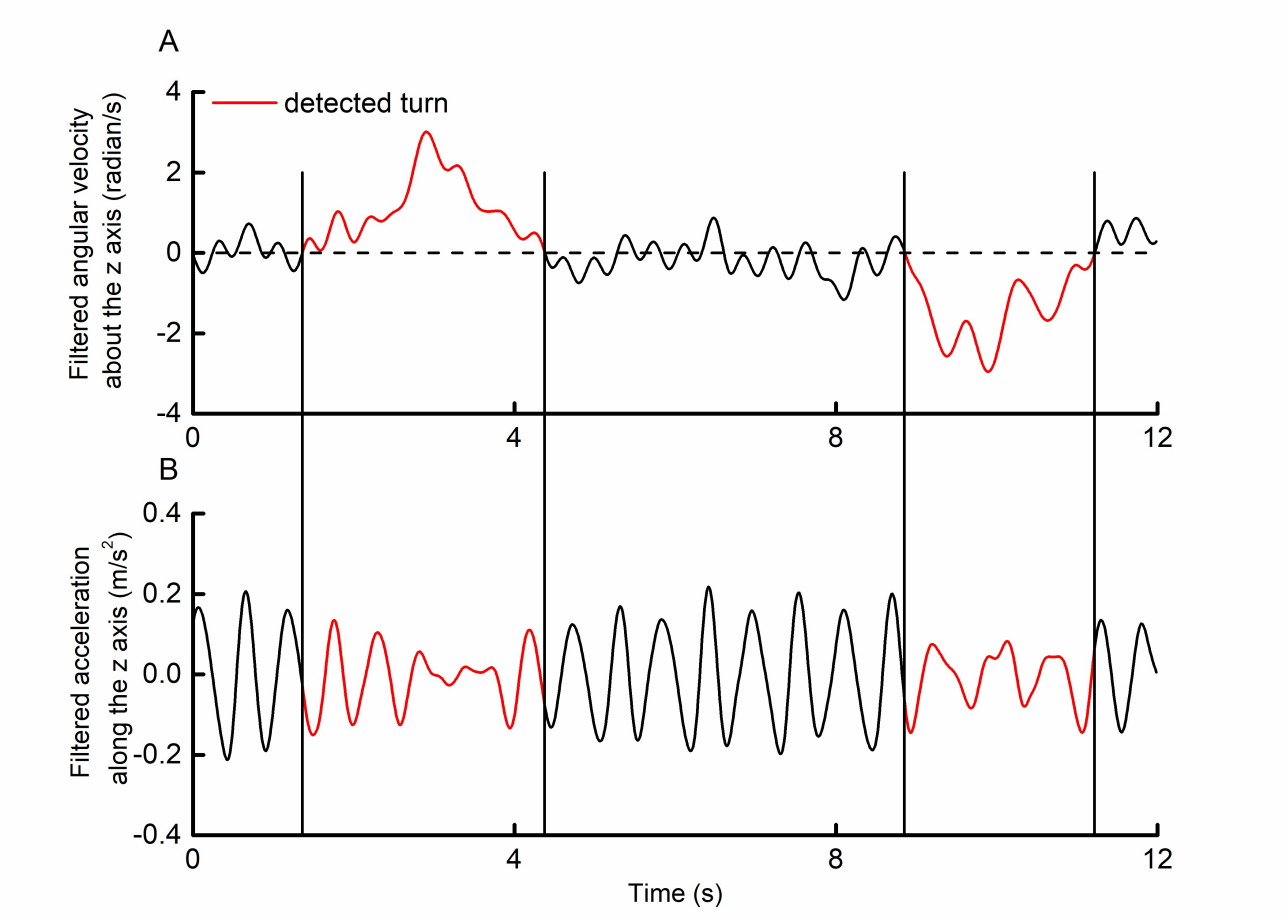
Example of smartphone-recorded (A) angular velocity and (B) acceleration relative to the earth coordinate system vertical axis during in-home walking with 2 detected periods of turning. Turns were identified from angular velocity time series and defined as any period between 2 consecutive zero crossings in which the product of the area under the curve and the time between zero crossings was >2.00 radian-seconds. Acceleration patterns were noticeably different during these periods. Average stride times from home assessment trials were thus computed from stride times derived only from nonturning periods.

Figure 4 illustrates an example of 2 identified turns within a selected trial of walking within the home. For all home walking trials, we first detected all potential turns as described above. We then calculated stride times from all z-axis acceleration data that occurred outside of detected periods of turning (using the same methods as those described in the “Laboratory Assessments” subsection.

### Statistical Analysis

We performed the following analyses with JMP Pro 13 (SAS Institute) and R version 3.3.1 (R Foundation). We set the significance level for all tests to *P*<.05.

#### Validity

We examined the validity of app-derived stride times by first assessing their agreement with corresponding stride times derived from the GAITRite mat using a Passing-Bablok orthogonal regression model, an appropriate approach for comparing methods while acknowledging measurement error [21]. Models included every stride that occurred during the first pass over the mat, for all trials of both normal and dual-task walking for each participant. We further examined the relationship between individual stride times derived from each device using linear regression and included visit (laboratory visits 1 and 2), task condition (normal or dual-task walking), and pocket tightness (tight, medium, or loose) as model effects to determine whether these factors influenced the observed relationship between derived stride times. In this model, we included participant as a random effect variable, as each contributed multiple data points to the model. We also used similar orthogonal and linear regression models to assess the agreement between dual-task costs to stride time as measured by the app and the GAITRite mat. We calculated cost from each pair of normal and dual-task walking trials as the percentage change (ie, increase) in average stride time.

For each individual stride that occurred on the GAITRite mat, we then calculated the magnitude of error between its stride time as calculated by the app and the GAITRite mat. We produced a Bland-Altman plot of this error to visualize this error as a function of stride time (ie, the average of the individual stride time as calculated by the app and the GAITRite mat). We used 2-way ANOVA to determine whether the magnitude of error, at the individual stride level, was influenced by task condition or pocket tightness.

#### Reliability

We examined the test-retest reliability of the app assessment by computing several intraclass correlation coefficients (ICCs). We calculated ICCs separately for normal and dual-task walking trials, for each of the following 4 conditions: (1) across trials within each laboratory assessment, (2) across trials over the 3 home assessments, (3) between the 2 laboratory visits, and (4) between laboratory and home assessments. For conditions 1 and 2, the unit of interest was the average stride time derived from each trial (ICC 1, 1). For conditions 3 and 4, the unit of interest was the average stride time, averaged across all trials of the same condition (ie, normal or dual task; ICCs 1, 3). We took ICC values greater than .80 to reflect excellent test-retest reliability.

#### Effects of Participant Characteristics, Walking Condition, and Testing Setting

We used Pearson correlations to examine relationships between average stride times and participant height and body mass. We used 2-way ANOVAs to examine the effects of walking condition (normal walking, dual tasking), setting (laboratory, home), and their interaction on average stride time. Significance level was set to *P*<.05.

## Results

We recruited 14 healthy participants aged 22 to 35 years (8 female; mean age 29.6, SD 4.2 years; mean height 168, SD 12 cm; mean body mass 76, SD 14 kg). All 14 participants completed both laboratory visits and all 3 home assessments. Across all 69 recorded assessments, 10 were completed with self-report of loose-fitting pockets, 24 with tight-fitting pockets, and 35 with pockets of medium tightness. For the 41 assessments completed at home, 11 were conducted in the morning, 13 in the afternoon, and 17 in the evening. Across participants, the average day-to-day variation in the range of timing of the 3 home assessments was 4.0 (SD 4.0) hours. The average number of strides detected in each 45-second home-based trial, after removal of turns, was 21.6 (SD 6.4) (range 13-28 strides).

### Validity of Smartphone-Derived Stride Time

**Figure 5:**
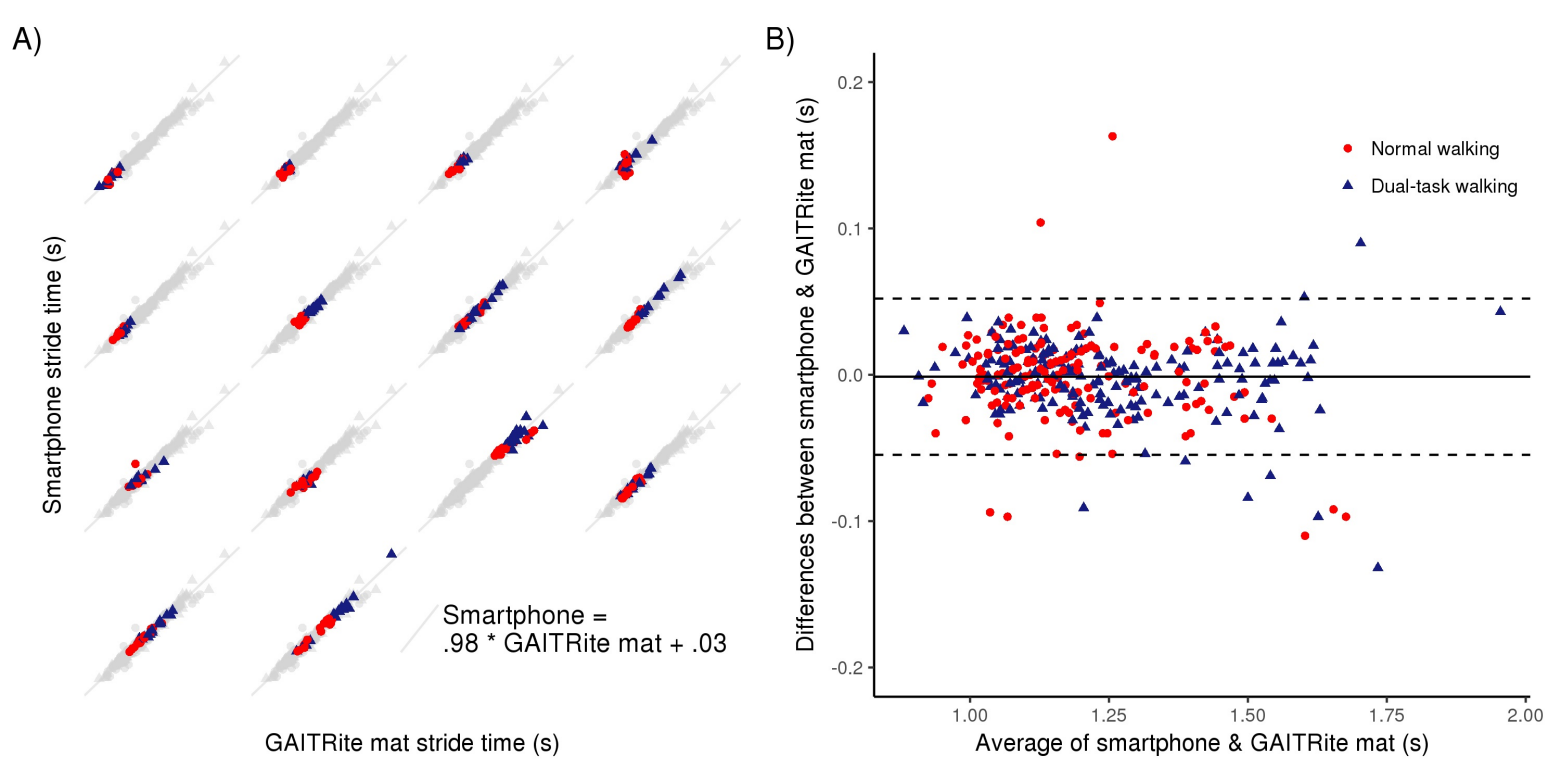
Correlation and agreement between stride times derived from a smartphone placed in the pocket and a GAITRite mat. (A) The timing of each individual stride that occurred over the GAITRite mat during all trials of normal and dual-task walking over 2 visits are presented separately for each participant. Stride times were noticeably longer during dual-task walking than during normal walking, for multiple participants. The gray background plot in each subplot is the same and represents the entire sample of stride times. Stride times derived from the app and the GAITRite mat were strongly correlated with one another (*r*^2^=.98, *P*<.001). The orthogonal best fit line of this entire sample had a slope of approximately 1 and an intercept of approximately 0. (B) Bland-Altman scatterplot depicting the difference (error) in measured time, as a function of the average time, for each stride as derived from the app and GAITRite mat.

For each detected stride across all participants and laboratory trials, stride times derived from the app demonstrated excellent validity as compared with the GAITRite mat. Orthogonal regression analysis revealed that stride times derived from the app were highly correlated with those measured by the GAITRite mat (*P*<.001, *r*^2^=.98; Figure 5).

**Figure 6:**
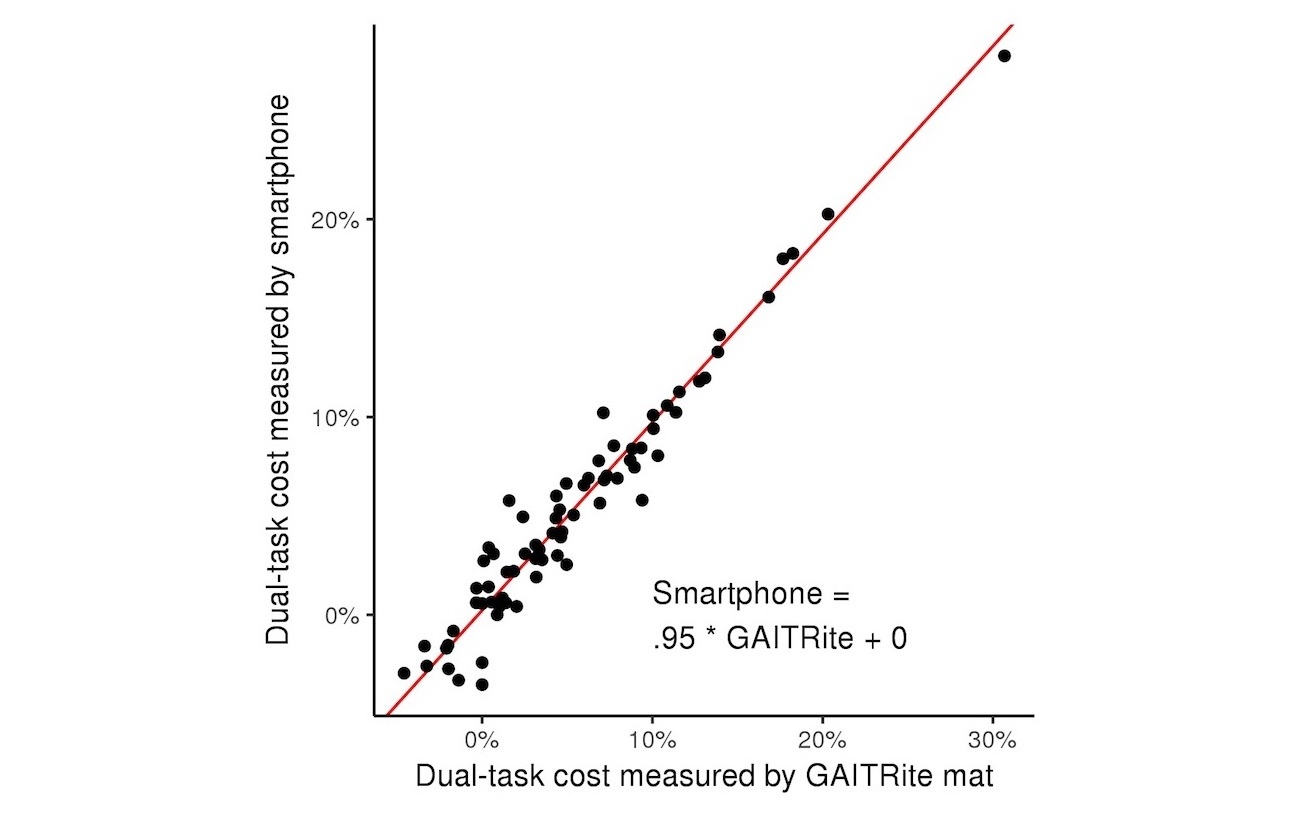
Relationship between dual-task costs to stride time as derived from a smartphone placed in the pocket and from a GAITRite mat. Dual-task cost was defined as the percentage change in average stride time derived from each pair of normal and dual-task walking trials. Dual-task costs as measured by the app and the GAITRite mat were strongly correlated with one another (*r*^2^=.95, *P*<.001). The orthogonal best fit line of these data had a slope of approximately 1 and an intercept of 0.

**Table 1:** Test-retest reliability of laboratory- and home-based assessments of average stride times during normal and dual-task walking.

Tests			Normal walking			Dual-task walking		
			ICC^a^	*P* value	95% CI	ICC	*P* value	95% CI
**GAITRite mat**								
	Within-visit		.94	<.001	.88-.98	.89	<.001	.77-.95
	Between-visit		.93	<.001	.79-.97	.83	<.001	.52-.94
**Smartphone app**								
	**Laboratory assessment**							
		Within-visit	.94	<.001	.88-.98	.90	<.001	.79-.96
		Between-visit	.92	<.001	.77-.97	.83	<.001	.51-.94
	Home assessment		.83	<.001	.62-.94	.82	<.001	.60-.94
	Laboratory vs home		.87	<.001	.57-.96	.89	<.001	.65-.97

^a^ICC: intraclass correlation coefficient.

Linear regression models further indicated that this correlation was unaffected by task condition, laboratory visit number, or self-report of pocket tightness.

The dual-task costs to average stride time derived from the app and the GAITRite mat were also highly correlated (*P*<.001, *r*^2^=.95; Figure 6). This correlation was also independent of laboratory visit number and self-reported pocket tightness.

The average magnitude of error of individual app-derived stride times, as compared with the corresponding stride time derived from the gait mat, was 16.9 (SD 9.0) ms. Figure 5 (part B) depicts a Bland-Altman plot, which illustrates that the magnitude of error was not noticeably influenced by stride time. ANOVA models further indicated that the magnitude of error was similar across laboratory visits (*F*_1346_=0.24, *P*=.63) and was unaffected by either walking conditions (*F*_1346_=0.03, *P*=.86) or pocket tightness (*F*_2346_=0.91, *P*=.40).

### Reliability of Smartphone-Measured Stride Time

Average stride times derived from each app trial—for both normal and dual-task walking—demonstrated excellent test-retest reliability across repeated trials within laboratory assessments, across trials between the 2 laboratory assessments separated by 1 week, and across home assessment days (Table 1). In general, we observed that ICC values were (1) slightly higher for trials of normal walking than for dual-task walking, (2) similar in value between app- and GAITRite-based measurements within each laboratory visit and between 2 laboratory visits, and (3) similar in value for home assessments and for laboratory assessments.

### Effects of Participant Characteristics, Walking Condition, and Setting on Stride Time

Average stride times were not significantly correlated with participant height or body mass. Stride times were longer (*F*_1158_=4.67, *P*=.03) when dual tasking (mean 1.18, SD 0.16 s) than when walking normally (mean 1.05, SD 0.16 s). Testing setting (ie, laboratory vs home) did not affect average stride times (*F*_1158_=0.001, *P*=.99).

## Discussion

This study provides a proof-of-concept in healthy adults that a smartphone placed in the front pocket of one’s pants or shorts can provide multimedia instructions to the participant and accurately measure stride times during walking under different experimental conditions. The app can detect major turns and compute average stride times during forward walking with high test-retest reliability within a laboratory or home setting.

Body-worn sensors, including those contained within smartphones, can be used to capture the kinematic properties of gait. Previous work has typically secured the smartphone or sensor tightly to the individual’s trunk [11,22-24] or lower extremities [10,25,26]. While that approach has been proven to enable measurement of gait metrics with enough sensitivity to distinguish between disease states, it has used additional equipment (eg, Velcro or elastic straps) together with trained personnel in a laboratory setting to provide assessment instructions. Our approach places the phone in the pocket, provides automated instructions to the participant, and uploads acquired data automatically via Wi–Fi to cloud-based storage, thus providing a widely accessible and cost-effective tool for the assessment of walking within both laboratory and nonlaboratory settings. Such a tool may be particularly useful and cost effective for large-scale national or international studies of mobility by obviating the need for local research staff to instruct participants or apply special instruments.

Walking in everyday life is frequently conducted while executing cognitive tasks. Serial subtraction is most typically used within laboratory dual-task paradigms because it is easily implemented, disrupts the gait of even healthy adults (see Figure 5, part A), and produces measurable dual-task costs that are sensitive to concussion [27,28], aging [20,29], future falls [30,31], and cognitive decline [32,33]. Dual-task assessments are influenced by the instructions provided to the participant prior to each trial of walking, especially with respect to task prioritization [34,35]. In this study, use of the smartphone to provide standardized instructions via a combination of animated, written, and audible instructions led to excellent test-retest reliability of average stride times derived from dual-task trials, both within the laboratory and at home, that were comparable with or even higher than published reports of similar assessments led by trained personnel [23,36]. Future work is therefore warranted to test and optimize this smartphone approach in older adults and those with varying levels of cognitive and physical impairment. Moreover, efforts are needed to use smartphone voice recognition software to quantify serial-subtraction performance in order to adjust dual-task cost outcomes for this important variable and to standardize cognitive-task difficulty across individuals.

The proposed method of identifying periods of walking that likely included turning, and subsequently removing these periods from the calculation of stride times, led to excellent test-retest reliability of average stride time. It is expected that the future development and application of more sophisticated turn identification algorithms will further improve test-retest reliability of this and other metrics by ensuring that strides included in subsequent analyses were not influenced by turning. Moreover, turning is critical to the navigation of one’s environment, and the kinematic characteristics of turning indeed provide important insight into the integrity of the locomotor control system [37-39]. Such an approach that leads to the accurate detection of a turn during remote walking assessments would also be highly valuable by enabling assessment of the kinematic properties of the turn itself.

This study has provided evidence that stride timing—a clinically meaningful outcome of locomotor control—can be accurately and reliably derived from kinematic data acquired by the smartphone when placed in the pocket when walking. Future work is warranted to establish the capability of this app to derive other clinically meaningful metrics of locomotor control, such as walking speed, swing and stance timing, or stride-time variability [17,40,41]. Finally, this study was focused on demonstrating the feasibility of assessing gait remotely using a smartphone and was thus completed in a relatively small cohort of healthy young adults. Larger studies are now needed to establish the validity and reliability of this method in more heterogeneous populations and in those with abnormal gait patterns.

## Abbreviations

AUC: area under the curve
ICC: intraclass correlation coefficient
